# Implementation of a Leave-behind Naloxone Program in San Francisco: A One-year Experience

**DOI:** 10.5811/westjem.2022.8.56561

**Published:** 2022-10-31

**Authors:** Kathy T. LeSaint, Juan Carlos C. Montoy, Eric C. Silverman, Maria C. Raven, Samuel L. Schow, Phillip O. Coffin, John F. Brown, Mary P. Mercer

**Affiliations:** * University of California, San Francisco, Department of Emergency Medicine, San Francisco, California; † San Francisco Fire Department, San Francisco, California; ‡ San Francisco Department of Public Health, San Francisco, California

## Abstract

**Introduction:**

In response to the ongoing opioid overdose crisis, US officials urged the expansion of access to naloxone for opioid overdose reversal. Since then, emergency medical services’ (EMS) dispensing of naloxone kits has become an emerging harm reduction strategy.

**Methods:**

We created a naloxone training and low-barrier distribution program in San Francisco: Project FRIEND (First Responder Increased Education and Naloxone Distribution). The team assembled an advisory committee of stakeholders and subject-matter experts, worked with local and state EMS agencies to augment existing protocols, created training curricula, and developed a naloxone-distribution data collection system. Naloxone kits were labeled for registration and data tracking. Emergency medical technicians and paramedics were asked to distribute naloxone kits to any individuals (patient or bystander) they deemed at risk of experiencing or witnessing an opioid overdose, and to voluntarily register those kits.

**Results:**

Training modalities included a video module (distributed to over 700 EMS personnel) and voluntary, in-person training sessions, attended by 224 EMS personnel. From September 25, 2019–September 24, 2020, 1,200 naloxone kits were distributed to EMS companies. Of these, 232 kits (19%) were registered by EMS personnel. Among registered kits, 146 (63%) were distributed during encounters for suspected overdose, and 103 (44%) were distributed to patients themselves. Most patients were male (n = 153, 66%) and of White race (n = 124, 53%); median age was 37.5 years (interquartile range 31–47).

**Conclusion:**

We describe a successful implementation and highlight the feasibility of a low-threshold, leave-behind naloxone program. Collaboration with multiple entities was a key component of the program’s success.

## INTRODUCTION

The United States is in the midst of an ongoing overdose epidemic. Since the late 1990s, the use of opioids in the US has accelerated to an unprecedented scale, and over the last decade the use of prescription and non-prescription opioids, prevalence of opioid use disorders, and opioid-related mortality have increased dramatically.^1^,^2^ In recent years, the steep increase in overdose deaths from prescription and illicit opioid use has been attributed to the rapid proliferation of illicitly made fentanyl and other highly potent synthetic opioids.

Since 1996 an increasing number of community-based programs have offered opioid overdose prevention services and provided the opioid antagonist naloxone hydrochloride to persons who use drugs, their families and friends, and to emergency medical services (EMS) personnel.^3^ By 2012, the training and provision of naloxone to 53,032 individuals had led to 10,171 drug overdose reversals.^3^ On April 5, 2018, the Surgeon General of the US Public Health Service released a health advisory urging the expansion of the use of and access to naloxone, making its widespread availability a key part of the nation’s public health response.^4^ Since then, more naloxone is being administered by law enforcement, EMS personnel, and non-emergency first responders to reverse opioid overdoses.^5^

Around the same time, federal and state regulations eased restrictions regarding distribution of naloxone to the public. In 2018 North Carolina became one of the first states to have an EMS-based naloxone leave-behind program in which emergency medical technicians (EMT) and paramedics leave naloxone with patients who decline transport to the emergency department.^6^ Subsequently, many EMS systems followed suit. In this manuscript, we describe a successful implementation of a city-wide, low-threshold. leave-behind naloxone program in San Francisco (SF), with collaboration between the county’s EMS base hospital, local and state EMS agencies, and the San Francisco Department of Public Health.

## METHODS

### Project Implementation

The project leadership team received funding from the Substance Abuse and Mental Health Services Administration to develop a naloxone training and low-barrier distribution program in SF called Project FRIEND (First Responder Increased Education and Naloxone Distribution). Direct costs for the first year of the project totaled approximately $300,000 and consisted of clinician salary support for the project director, lead project developer, lead evaluator, and curriculum lead; the hiring of a full-time project coordinator; and the hiring of two content-expert consultants, one in EMS and the other in substance use disorders. During program design, the team also assembled a voluntary advisory committee composed of leaders from the SF EMS Agency, leaders from each of the three SF 9-1-1 ambulance provider organizations, a harm-reduction expert from the Drug Overdose Prevention and Education (DOPE) Project, and a clinical pharmacist. San Francisco’s three 9-1-1 public and private entities that provide ambulance service include the SF Fire Department, King-American Ambulance Company, and American Medical Response, Inc. In addition, the team sought out an EMT or paramedic representative from each of the three SF 9-1-1 ambulance services to serve as volunteer, Project FRIEND “champions” for peer-based support.

Since inception, the leadership team met twice monthly to develop protocols and track progress for program implementation milestones. The leadership team, consultants, advisory committee, and champions met quarterly during the first year to finalize deliverables prior to implementation and then had semi-annual formal meetings after. In collaboration with local and state EMS agencies, the team augmented existing protocols and policies to allow for naloxone distribution by prehospital personnel, created in-person and virtual training curricula for SF EMS personnel, and developed an evaluation and data collection tool for the naloxone kits that were distributed. The program’s workflow and educational curriculum presentation are included as supplementary material (Appendices 1 and 2). Prior to the start of the project, the team applied for and received intranasal naloxone hydrochloride kits from the California Department of Health Care Services under the Naloxone Distribution Project.^7^ The team received two separate shipments from the Naloxone Distribution Project, totaling 1,200 kits.

**Population Health Research Capsule**

What do we already know about this issue?

*In response to the ongoing US opioid epidemic, an increasing number of emergency medical services (EMS)-based, leave-behind naloxone programs have been implemented*.

What was the research question?

*How did a project team implement a low-threshold, leave-behind naloxone program in San Francisco?*

What was the major finding of the study?

*The implementation of an EMS-based, leave-behind naloxone program is feasible in an urban setting. Of 1,200 kits given to EMS, 19% were distributed to patients or bystanders in the field*.

How does this improve population health?

*An EMS-based, leave-behind naloxone program can be complementary to other harm reduction efforts*.

On September 11 and November 13, 2019, the leadership team hosted in-person, formal training sessions, offered to all EMS personnel in SF. The training sessions detailed Project FRIEND’s background and goals, indications and instructions for use of naloxone, “just-in-time” training on methods for educating patients/bystanders in naloxone administration, and an overview of harm reduction strategies. These in-person trainings lasted approximately 60 minutes and included a slide-deck presentation and question-and-answer session. In addition, the team hosted 12 just-in-time training sessions during the first two months of project implementation. For each just-in-time session, a project team member was present at each of the three 9-1-1 ambulance provider organizations during EMS shift changes to conduct informal, verbal training sessions to available personnel and to distribute quick response (QR) code website links to additional training materials. These just-in-time training sessions lasted approximately 10–20 minutes including a question-and-answer session.

### Data Collection Process

Each naloxone intranasal kit was labeled with a Project FRIEND serial number and QR code for registration and data tracking, as well as the Project FRIEND website URL to direct recipients to additional information and community resources. Every three months, approximately 100–200 kits were delivered to the operations manager at each of the three EMS organizations for allocation to individual ambulances/EMS personnel.

San Francisco’s EMS personnel were asked to register and distribute naloxone kits to any individuals (patient or bystander) they deemed at risk of opioid overdose or likely to come into contact with a high-risk individual. These encounters could take place at any time while on shift and were not limited to overdose calls. The QR code linked to a secure online survey (Qualtrics LLC, Provo, UT) in which paramedics and EMTs entered the following information: kit serial number; whether EMS specifically responded to an overdose event; the location of the naloxone distribution; EMS personnel identifiers (name, ambulance company, EMS incident number, contact information for the EMT or paramedic); and naloxone recipient demographic information (name, age, gender, race). Registration of each kit was optional; during the training sessions, we prioritized the goal of low-barrier distribution and patient care/rapport over strict adherence to data collection.

For each naloxone kit that was distributed, the EMT or paramedic who registered the kit was entered into a monthly lottery for a $100 gift card incentive. During the first year of program implementation, the leadership team created advertisements and conducted outreach to increase participation by EMTs and paramedics. Outreach materials included quarterly newsletters, reminder emails, and personnel-facing signage posted in each ambulance. Additionally, near the completion of the first year of implementation, the program team hosted a webinar on EMS opioid management for the EMS and healthcare community. Speakers included national and regional leaders in EMS-based harm reduction programs and featured a front-line worker presentation from one of Project FRIEND’s paramedic champions.

## RESULTS

A video training module was distributed to over 700 EMS personnel at each of SF’s three 9-1-1 ambulance provider organizations. Two-hundred twenty-four (32%) EMS personnel were trained by the Project FRIEND team: 29 attended one of two larger in-person training sessions, and 195 were captured during one of 12 just-in-time training sessions. From September 25, 2019–September 24, 2020, a total of 1,200 Project FRIEND QR-labeled naloxone kits were distributed to three SF EMS organizations. Kits were distributed to each of these organizations on five occasions throughout the year, per each organization’s request after they had exhausted or nearly exhausted their supply. Of these, 232 (19%) were registered over 12 months from September 25, 2019–September 24, 2020. During this time, an average of 19 kits were registered per month, with the highest number of registered kits (n = 56) recorded during the first month of project implementation. We did not observe a relationship between the number of registered kits and the distribution of advertisements and outreach materials.

In 146 of 232 registered kits (63%), EMS personnel had been dispatched to an overdose event. Most naloxone kits were distributed in the Downtown/Civic Center neighborhood of SF (n = 96, 41%). Many were directly given to patients (n = 103, 44%), a majority of whom were male (n = 153, 66%), of White race (n = 124, 53%), and with a median age of 37.5 years (interquartile range 31–47). Other naloxone kits were distributed to bystanders (n = 77, 33%), or to a friend/family member (n = 38, 16%); in 14 cases (16%) this data was unknown.

Having observed a decrease in the number of kits registered in the second month of the project (nine kits distributed), we conducted a focus group with our three EMS champions and leadership from ambulance organizations to discuss barriers to distribution and reporting in December 2019. Additionally, we performed a formal program data overview for the SF EMS Agency as well as a systemwide quality review at the six-month program mark. Prior to these reviews, we asked the EMS champions and agency leadership to conduct informal conversations with their colleagues regarding operational challenges to the Project FRIEND program. In these discussions, we found that EMS personnel either did not remember to offer naloxone or had already exhausted their stock but otherwise did not report barriers to distribution. Barriers to registering kits reported included lack of time (eg, having to respond to another prehospital incident immediately) and being unfamiliar with the registration protocol (eg, did not watch the training video). We also learned that the program became known to individuals not treated by EMS; anecdotally, EMTs and paramedics described several instances in which they were approached by passers-by (ie, non-patients) for naloxone kits.

## DISCUSSION

We found a low barrier, EMS-based naloxone leave-behind program to be feasible in an urban setting. While the leadership team involved in the creation of this program was federally funded, the program itself required few start-up resources. Many of those on the project development team (advisory committee, EMS champions) were available on a voluntary basis, as the goals of Project FRIEND were aligned with growing interest in prehospital opioid harm reduction. While implementing the program was time intensive, it had low direct cost (eg, email for communication, use of printing services). To further reduce costs, our program used the California Department of Health Care Services’ Naloxone Distribution Program to obtain naloxone intranasal kits at no cost. As interest in harm reduction strategies continues to increase throughout the US, we encourage those seeking to create a leave-behind program to take advantage of similar state and federally funded naloxone supply programs.

A majority of the naloxone kits were distributed in SF’s Downtown/Civic Center area. This neighborhood is one of the city’s poorest, containing the highest number of single residence occupancy hotels (which are known to be associated with a higher overdose mortality) and many marginally or unhoused people.^8^ While many of the existing SF harm reduction programs are located in and concentrate their efforts on this neighborhood, the patients most likely to use existing programs are those with prior overdose experiences, who use multiple substances and who are unhoused.^9^ Our program aimed to fill the existing gap by providing naloxone to high-risk individuals who have overdosed, regardless of their housing status, use of other substances, or experience with prior overdoses. In addition, nearly one third of the naloxone kits were distributed outside the Downtown/Civic Center area, suggesting a sizable need for harm reduction and substance use treatment resources in other parts of SF.

While communities differ greatly in terms of the types of opioids used, the populations of patients most at risk of overdose, and the community resources available to address opioid overdose, our setting has many features shared by other communities. As in other communities, heroin had been the primary opioid driving opioid overdose mortality for decades before being overtaken by prescription opioids in the mid-2000s; use of heroin then increased above prescription opioid use until fentanyl became the cause of the vast majority of overdoses.^10^ A study from of opioid overdose deaths in SF from 2009–2019 revealed an upward trend in fentanyl-related fatal accidental overdoses.^11^ In addition, a comprehensive study of out-of-hospital cardiac arrest deaths in SF demonstrated that more than 1 in 6 resulted from an occult overdose, suggesting the scope of the overdose epidemic is worse than previously thought.^12^ The city’s EMS personnel have reported increases in the number of patients requiring naloxone for opioid overdose reversal in recent years, further attesting to the rising opioid problem in SF. The SF Department of Public Health (reported 365 lay overdose reversals with naloxone in 2014. However, in 2015 the number of reversals rose sharply to 980, nearly doubled again in 2018 to 1,658, and rose to 4,307 in 2020.^10^

During the first year of Project FRIEND implementation, a majority of registered kits did not go directly to patients, but rather to bystanders, and to friends or family of patients. This is particularly important, as involvement of a patient’s support system is critical to the addiction treatment process.^13^,^14^ Training and equipping the patient’s close contacts with naloxone, a life-saving tool, may be the first step in opening a dialogue for long-term treatment. Additionally, family members and close support systems are deeply affected by an individual using substances and may suffer high levels of distress, family conflict, domestic violence, unmet social needs, and economic burdens.^15^ Involving the patient’s close contacts in addiction treatment has been shown to increase entry into treatment and enhance treatment completion, and has been linked with improved treatment outcomes for the individual coping with addiction.^16^

EMS-initiated interventions for substance use disorders are critical, and paramedics and EMTs can play a significant role in the public health sphere. While a low-barrier naloxone kit distribution initiative does not equate to provision of comprehensive substance use disorder treatment, it does represent a step toward management of substance use and related risks.^17^ EMS-based interventions that more comprehensively treat patients with substance use disorders have only recently emerged and range from those that provide medications for substance use disorders (eg, buprenorphine) to those focused on addressing social determinants of health.^18^–^20^ We anticipate that these initiatives will continue to be systematically studied, and our hope is to expand Project FRIEND to include additional evidence-based care strategies in the near future.

The main challenge our leave-behind naloxone program faced was buy-in by EMS personnel regarding data tracking. While the project team had the support of the SF EMS Agency and local EMS organizations, the program depended on the willingness of individual EMTs and paramedics to engage patients and bystanders to participate in the Project FRIEND data collection tool. While the number of kits distributed was substantially greater than the number actually registered, we did not formally evaluate why specific kits went unregistered or why kits may not have been distributed during particular encounters. Because the primary goal of this project was to distribute naloxone, we opted for a low-barrier mechanism with an optional registration process. While a system with mandatory data elements might have yielded more robust results, it would likely also have resulted in less naloxone being distributed. Notably, we previously reported that EMTs and paramedics in SF generally believe that naloxone distribution programs are effective and they support the training and distribution being performed by prehospital personnel.^21^

## LIMITATIONS

Several limitations of our program warrant consideration. First, as mentioned previously, we had low adherence to the voluntary kit-registration process and were only able to obtain demographic data on a small proportion of the total kits distributed. Improvement in data-tracking/kit registration may have been accomplished through provision of incentives, ongoing active outreach to EMS personnel, or making the registration of kits compulsory prior to distribution. However, the primary purpose of the program was not to collect data nor to conduct a formal research study. While demographic data may help identify high-risk individuals or the locations of greatest need for harm reduction, the goal of the program was to introduce low-barrier naloxone distribution through SF EMS. During the first year of Project FRIEND implementation, 1,200 naloxone intranasal kits were distributed to members of the SF community by prehospital personnel, in some instances when EMS was not even responding to an overdose event.

Project FRIEND had a first-year direct cost of $300,000, an amount that may not be easily obtainable by EMS groups wishing to start a similar program. At the time of Project FRIEND inception, however, we were one of very few naloxone leave-behind programs in the country and, thus, required clinician and consultant expertise during our start-up phase. Currently, many more EMS-based naloxone leave-behind programs are in existence. EMS groups in the start-up phase now have more opportunities for collaboration with existing programs and experts.

Furthermore, our program did not follow individual patients over time; therefore, we were unable to determine whether the distributed naloxone was used to reverse a future overdose event if it was not reported. In addition, while we provided a link to the Project FRIEND website on each kit, we acknowledge that not all patients have access to smartphones or the internet; thus, we were unable to determine how many patients subsequently sought treatment for opioid use disorder or had linkage to care. Finally, it was not possible for us to determine the direct long-term implications of our leave-behind naloxone program, such as healthcare system cost-effectiveness or changes to morbidity and mortality related to opioid use disorder.

## CONCLUSION

We highlight the feasibility of implementing a low barrier, EMS-based naloxone leave-behind program. While this initiative was created in San Francisco, an urban area, the basic premise for implementation of this type of program is generalizable to other communities. Because harm reduction is often the first step in the treatment of high-risk individuals with opioid use disorder, enlisting EMS personnel to engage these individuals is a promising method to help save lives and reduce future overdose events.

## Supplementary Information




