# Supplementary material for: Implementation of a Leave-behind Naloxone Program in San Francisco: A One-year Experience
Source: West J Emerg Med. 2022 Oct 31;23(6):952–7. doi: 10.5811/westjem.2022.8.56561 (PMC9683755; doi:10.5811/westjem.2022.8.56561)
Supplement: Supplementary file 1 [file wjem-23-952-s001.docx]

**Implementation of a leave-behind naloxone program in San Francisco:
A one-year experience**

**Appendix 1**

**San Francisco EMS Program Workflow for EMS-Distributed Project FRIEND Naloxone**

**Patient Assessment – Screening for Project FRIEND**

Patients presenting with any one of the following conditions are eligible for Project FRIEND Naloxone Distribution:

· Opioid overdose (requiring naloxone administration or supportive care and monitoring)

· History or physical exam with evidence of illicit drug use or paraphernalia (e.g. history of intravenous drug use, track marks, needles present in belongings, etc.)

· History or physical exam with prescription opioid use (prescribed or recreational)

· Physical environment with illicit, multiple, or high-dose prescription opioids present

**Project FRIEND Distribution Workflow**

· Assess patient decision-making capacity

· Offer leave behind naloxone with just-in-time training to patient and/or appropriate bystander(s)

o Perform teaching and direct recipient to visual aids on naloxone kit

o Distribute naloxone kit with Project FRIEND and OBIC contact

o Register distribution with Project FRIEND by scanning QR code and follow prompts (preferred method), OR

o Record distribution on Project FRIEND log

o Contact the Base Hospital if

§ Patient requesting to AMA (and no second paramedic available or physician consultation is desired)

**End of Shift Procedures**

o Re-stock ambulance with Project FRIEND naloxone kits

o Turn in completed log to Project FRIEND Collection Box

**Community Outreach Training and Distribution Events**

Once a paramedic or EMT has participated in a TTT program, he or she may serve as a Project FRIEND trainer and assist in community outreach and education public health and safety events. He or she may distribute and educate the public about naloxone at these events.

o Perform teaching and direct recipient to visual aids on naloxone kit

o Distribute naloxone kit with Project FRIEND and OBIC contact

- Register distribution with Project FRIEND by scanning QR code and follow prompts (preferred method)
