# Supplementary material for: Implementation of a Leave-behind Naloxone Program in San Francisco: A One-year Experience
Source: West J Emerg Med. 2022 Oct 31;23(6):952–7. doi: 10.5811/westjem.2022.8.56561 (PMC9683755; doi:10.5811/westjem.2022.8.56561)
Supplement: Supplementary file 2 [file wjem-23-952-s002.pdf]

Implementation of a leave-behind naloxone program in San Francisco:
A one-year experience

Appendix 2. Slide deck presentation of Project FRIEND project implementation

ProjectFRIEND
FIRST RESPONDER INCREASED EDUCATION AND NALOXONE DISTRIBUTION

1

Overall Mission & Goals

- Reduce opioid related deaths through prevention, treatment, and recovery activities in San Francisco
- **Goal: to increase naloxone utilization in San Francisco**
  - Training of first responders
  - Training in naloxone administration and distribution
- **Goal: to increase referrals and resource utilization for patients with opioid use disorder**
  - Contact & give resources to all patients either *administered* or *distributed* naloxone by certified first responders
  - Increase access to treatment programs
- Good for patients, good for providers, no cost, low time commitment

2

Partners/Sponsors

- SF Fire Department
- American Medical Response
- King American
- Office Based Induction Clinic
- Syringe Access Services
- SF AIDS Foundation
- And many more…

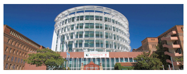

3

Objectives

- Recognize risk factors for opioid overdose
- Identify an overdose and indications for naloxone
- How to respond to an overdose
- How/when to distribute naloxone
- How to train others to use naloxone
- Questions

4

Case 1

5

Experience

- Who has witnessed/treated an opioid overdose?
- What went well/could be improved in Case #1?
- Do you think we are making a difference?

6

Opioid-related overdose mortality rate* San Francisco, 2010-2015

ProjectFRIEND
FIRST RESPONDER INCREASED EDUCATION AND NALOXONE DISTRIBUTION

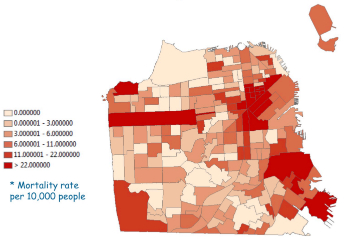

- Patients receiving naloxone:
  - San Francisco Fire Dept:
    - 2018: 1,151
    - 2017: 772
    - 2016: 691
  - DOPE Project:
    - 2017: 1,257
  - **Project FRIEND:**
    - **2019: TBD**

7

Risk Factors for Overdose

ProjectFRIEND
FIRST RESPONDER INCREASED EDUCATION AND NALOXONE DISTRIBUTION

- Mixing drugs
  - Opioids + other sedative-hypnotic drugs/medications (alcohol, benzodiazepines, etc)
- Reduced tolerance
  - Frequent use
  - Rehab/detox
  - Jail/prison
  - Sickness/hospitalization
- Increased dependence
  - Chronic or acute pain
- New/different supply
- New route (IV instead of pills/snorting)
- Using alone or injection by partner

8

Identifying Opioid Overdose

ProjectFRIEND
FIRST RESPONDER INCREASED EDUCATION AND NALOXONE DISTRIBUTION

- Unresponsive
- Slowed or irregular respirations/apnea
- Pinpoint pupils
- Cyanosis
- Clammy skin
- Hypoxia

9

Opioid and Naloxone

Mechanism of Action

ProjectFRIEND
FIRST RESPONDER INCREASED EDUCATION AND NALOXONE DISTRIBUTION

Opioid receptor binding

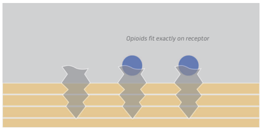

Naloxone: competitive antagonist

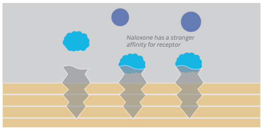

How long does naloxone last?

10

Treatment of Overdose

ProjectFRIEND
FIRST RESPONDER INCREASED EDUCATION AND NALOXONE DISTRIBUTION

- Initial actions:
  - Assess scene safety
  - Verbal/tactile stimulation
  - Reposition airway
  - BVM as needed
- Indications for naloxone:
  - Apnea
  - Hypoxia
  - Inability to protect airway
  - Concern for dangerous co-ingestion (e.g. acetaminophen) or comorbid medical problems

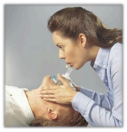

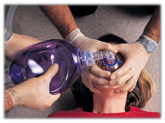

11

Considerations

ProjectFRIEND
FIRST RESPONDER INCREASED EDUCATION AND NALOXONE DISTRIBUTION

- Dose:
  - Range: 0.04mg –> 2mg –> 4mg –> more?
  - Available formulations: IV, IM, IN, IO
  - Give enough to resume spontaneous respirations +/- regain consciousness
    - If witnessed overdose/no other concerns, not necessary to completely reverse
    - May precipitate immediate withdrawal –> vomiting, confusion/aggression, severe pain, refusal of additional medical care
  - Pre-packaged intranasal naloxone (Narcan): 4mg
  - May need to re-dose

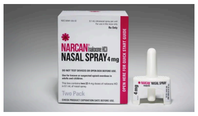

12

Eligibility for Project FRIEND Naloxone Distribution

ProjectFRIEND
FIRST RESPONDER INCREASED EDUCATION AND NALOXONE DISTRIBUTION

- Patients with **any** of the following conditions are eligible for Project FRIEND naloxone distribution:
  - Opioid overdose (requiring naloxone administration or supportive care and monitoring)
  - History or physical exam with evidence of illicit drug use or paraphernalia (e.g. history of IV drug use, track marks, needles present in belongings, etc.)
  - History or physical exam with prescription opioid use (prescribed or recreational)
  - Physical environment with multiple or high-dose prescription opioids present

13

After Naloxone Treatment

ProjectFRIEND
FIRST RESPONDER INCREASED EDUCATION AND NALOXONE DISTRIBUTION

- Supportive and caring environment
  - Common reactions: emergence reaction, fear of EMS or Police, past trauma
- Recommend transport to ED
- If patient refuses:
  - Assess medical decision making capacity (consider co-ingestions)
  - ZSFG Base Hospital contact (Case 1 MD response)
    - Patient requesting to AMA
    - Patient agrees to a follow-up call after ED visit/hospitalization or AMA
  - Educate: Naloxone only lasts up to 90 min –> can re-overdose –> death

14

ProjectFRIEND
FIRST RESPONDER INCREASED EDUCATION AND NALOXONE DISTRIBUTION

- Offer leave behind naloxone to patient and/or appropriate bystander(s)
  - Perform teaching and direct recipient to visual aids on naloxone kit
  - Distribute naloxone kit with Project FRIEND information
  - Register distribution with Project FRIEND by scanning QR code and following prompts
- Encourage safe use/harm reduction practices:
  - Use with friend, don't mix drugs, have naloxone ready, etc.

15

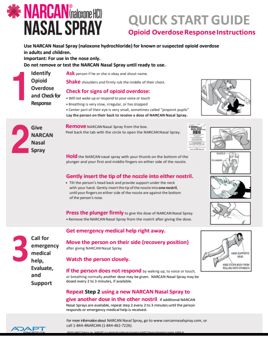
NARCAN® (naloxone HCl) NASAL SPRAY
QUICK START GUIDE
Opioid Overdose Response Instructions

Use NARCAN Nasal Spray (naloxone hydrochloride) for known or suspected opioid overdose in adults and children.
Important: For use in the nose only.
Do not remove or test the NARCAN Nasal Spray until ready to use.

1 Identify Opioid Overdose and Check for Response
Ask person if he or she is okay and shout name.
Shake shoulders and firmly rub the middle of their chest.
Check for signs of opioid overdose:
- Will not wake up or respond to your voice or touch
- Breathing is very slow, irregular, or has stopped
- Center part of their eye is very small, sometimes called "pinpoint pupils"

Lay the person on their back to receive a dose of NARCAN Nasal Spray.

2 Give NARCAN Nasal Spray
Remove NARCAN Nasal Spray from the box.
Peel back the tab with the circle to open the NARCAN Nasal Spray.
Hold the NARCAN nasal spray with your thumb on the bottom of the plunger and your first and middle fingers on either side of the nozzle.
Gently insert the tip of the nozzle into either nostril.
- Tilt the person's head back and provide support under the neck with your hand. Gently insert the tip of the nozzle into **one nostril**, until your fingers on either side of the nozzle are against the bottom of the person's nose.

Press the plunger firmly to give the dose of NARCAN Nasal Spray.
- Remove the NARCAN Nasal Spray from the nostril after giving the dose.

3 Call for emergency medical help, Evaluate, and Support
Get emergency medical help right away.
Move the person on their side (recovery position) after giving NARCAN Nasal Spray.
Watch the person closely.
If the person does not respond by waking up, to voice or touch, or breathing normally another dose may be given. NARCAN Nasal Spray may be dosed every 2 to 3 minutes, if available.
Repeat **Step 2** using a new NARCAN Nasal Spray to give another dose in the other nostril. If additional NARCAN Nasal Sprays are available, repeat step 2 every 2 to 3 minutes until the person responds or emergency medical help is received.

For more information about NARCAN Nasal Spray, go to www.narcannasalspray.com, or call 1-844-4NARCAN (1-844-462-7226).
ADAPT PHARMA

ProjectFRIEND
FIRST RESPONDER INCREASED EDUCATION AND NALOXONE DISTRIBUTION

16

ProjectFRIEND
FIRST RESPONDER INCREASED EDUCATION AND NALOXONE DISTRIBUTION

EMT / paramedic to complete when handing out leave-behind Narcan kits

Scan QR code (on box of each kit) →

1. Kit number
2. Were you on scene for an overdose?
3. Location of distributed naloxone
4. Who was the kit given to?
5. Recipient demographic information: name, sex, age/DOB, race/ethnicity
6. EMS personnel information: name, email, EMS company affiliation

17

End of Shift Procedures

ProjectFRIEND
FIRST RESPONDER INCREASED EDUCATION AND NALOXONE DISTRIBUTION

- Re-stock ambulance with Project FRIEND naloxone kits
  - Can ask your operations manager with assistance
- Register kits that were distributed during your shift

18

Legal Considerations

- Project FRIEND is a federally funded project by SAMHSA
- California state laws protect those administering and distributing naloxone
- San Francisco EMS Agency issued standing order for ALS/BLS administration and distribution
- Good Samaritan laws for bystanders
- Naloxone available without physician prescription (dispensed by pharmacists)

19

Review

- Case 2
- Would you administer naloxone?
- What are your other options?

20

Questions/discussion

21
